# Detection and quantification of bovine papillomavirus DNA by digital droplet PCR in sheep blood

**DOI:** 10.1038/s41598-021-89782-4

**Published:** 2021-05-13

**Authors:** Sante Roperto, Anna Cutarelli, Federica Corrado, Francesca De Falco, Canio Buonavoglia

**Affiliations:** 1 Dipartimento di Medicina Veterinaria e Produzioni Animali, Università degli Studi di Napoli Federico II, Via Veterinaria, 1, 80137 Naples, Italy; 2 Istituto Zooprofilattico Sperimentale del Mezzogiorno, Via della Salute 2, 80055 Portici, Naples, Italy; 3 Dipartimento di Medicina Veterinaria, Università degli Studi di Bari “Aldo Moro”, Strada Provinciale per Casamassima Km3, 70010 Valenzano, Bari, Italia

**Keywords:** Immunology, Molecular biology, Diseases, Pathogenesis

## Abstract

Highly pathogenic bovine papillomaviruses (BPVs) were detected and quantified for the first time using digital droplet polymerase chain reaction (ddPCR) by liquid biopsy in 103 clinically healthy sheep. Overall, ddPCR detected BPVs in 68 blood samples (66%). BPV infection by a single genotype was revealed in 61.8% of the blood samples, and BPV coinfection by double, triple or quadruple genotypes was observed in 38.2% of liquid biopsies. The BPV-2 genotype was most frequently seen in sheep, whereas BPV-1 was the least common. Furthermore, ddPCR was very useful for detection and quantification; the BPV-14 genotype was observed for the first time in ovine species, displaying the highest prevalence in some geographical areas (Apulia). In 42 of the positive samples (61.8%), a single BPV infection was observed, 26 of which were caused by BPV-2 (61.9%) and 7 by BPV-13 (16.7%). BPV-14 was responsible for 7 single infections (16.7%) and BPV-1 for 2 single infections (4.7%). Multiple BPV coinfections were observed in the remaining 26 positive samples (38.2%), with dual BPV-2/BPV-13 infection being the most prevalent (84.6%). BPV infection by triple and quadruple genotypes was also observed in 11.5% and 3.8% of cases, respectively. The present study showed that ddPCR, a biotechnological refinement of conventional PCR, is by far the most sensitive and accurate assay for BPV detection compared to conventional qPCR. Therefore, ddPCR displayed an essential diagnostic and epidemiological value very useful for the identification of otherwise undetectable BPV genotypes as well as their geographical distributions and suggesting that animal husbandry practices contribute to cross-species transmission of BPVs.

## Introduction

Papillomaviruses (PVs) comprise a diverse group of epitheliotropic, double-stranded DNA viruses that infect humans and animals in a species-specific manner^1^. PVs have co-evolved with their respective hosts, resulting in minimal cross-transfer between species^2^. Viruses such as PVs that slowly evolve with their hosts typically cause latent infection^3^. However, persistent infection of cutaneous and mucosal epithelia by PVs induces cellular proliferation^1^.

Bovine papillomaviruses (BPVs) comprise 29 genotypes^4,5^. Four highly pathogenic BPVs (BPV-1, -2, -13, and -14) belong to the *Delta* genus (δPVs)^6,7^. They are associated with both cutaneous and mucosal benign and malignant tumors. Indeed, BPV-2 and BPV-13 are the most notable infectious agents commonly responsible for bladder tumors in some breeds of pasture-residing cattle that graze on lands rich in bracken fern (Pteridium spp.)^8,9^.

δPVs are the only BPVs known to infect mesenchymal tissues and to show cross-species transmission and infection^1^. δPVs have been detected in cutaneous wart lesions from ovines^10,11^. Vertical transmission of δPVs in sheep, resulting in oral fibropapillomatosis and epidermal hyperplasia of the lip in newborn lambs, has also been documented^12^. Furthermore, δPV DNA has been detected by polymerase chain reaction (PCR) in the peripheral blood of healthy sheep^13^.

Although there are very limited numbers of reports describing BPV quantification data, PV studies have traditionally used real-time quantitative PCR (qPCR) to measure the virus reservoir represented by PV DNA in both cutaneous and bladder neoplastic samples^14–17^. Most recently, digital PCR is gaining popularity as a novel approach to nucleic acid quantification as it allows for absolute target quantification. Indeed, digital droplet PCR (ddPCR) is a robust PCR technique that enables precise and accurate absolute quantification of target molecules by diluting and partitioning the samples into numerous compartments^18^.

Quantification of PVs by digital PCR is proving to be a valuable improvement over qPCR, as it has been shown to have a higher robustness to mismatches between the primer-probe set and PV genotypes^7,19^. Due to pathogens that cause latent infection, BPV concentrations in the blood are sometimes too low to be determined by traditional methods. In cattle and goats, ddPCR has been found to outperform qPCR in terms of the sensitivity, specificity, and reproducibility of BPV detection, all of which play a central role in diagnostic and epidemiological procedures to identify the geolocalization of BPVs^7,19^.

The present study aimed to detect and quantify BPV DNA in the peripheral blood of sheep using ddPCR and to show the potential advantages of this molecular technology in the diagnosis and epidemiology of infectious diseases, including viral diseases.

## Materials and methods

### Liquid biopsy samples and DNA extraction

Blood samples from 103 apparently healthy 1- to 3-year-old sheep were collected from the jugular vein in vacutainers containing ethylenediaminetetraacetic acid (EDTA). All the examined sheep were clinically healthy as they had a regular milk production and a very good lambing activity. Furthermore, the official veterinarian responsible for health conditions of the flocks provided us the medical records of the animals showing that examined sheep did not have any therapeutic treatment for disease.

A total of 40 samples were obtained from sheep living in Sardinia (Sar) (20) and Campania (Cam) (20), 48 samples from Calabria (Cal) (24) and Basilicata (Bas) (24), and 15 samples from Apulia (Apu). Sheep flocks from Sar, Cam, Cal, Bas, and Apu were composed of 180, 190, 210, 250, and 120 animals, respectively. All sheep excluding those from Cam, were from flocks lived on pasturelands located at 800–1000 m asl altitudinal range; during the day sheep were free at pasture; at night they were grouped in fences adjoining cattle households. Sheep from all these flocks shared the bracken fern-infested highlands that they grazed on with pasture-residing cattle. Indeed, in highland areas, fern plant is the main food for herbivores which live free at pasture during all the seasons. In the dry season, grasses are wilted and there is a scarcity of fresh pastures, but bracken frond remain green and thus attractive to herbivores. Furthermore, rainy season allows adequate conditions for strong bracken growth. Sheep from Cam were from flocks living in lowland areas where bracken is virtually absent and in closed pens without any contact with other animals. Furthermore, sheep feeding of this flock was composed prevalently of fresh pastures from mixed meadows supplemented by concentrates and hay fodder. Total DNA was extracted using a DNeasy Blood & Tissue Kit (Qiagen, Wilmington, DE, USA), according to the manufacturer’s instructions.

### qPCR

qPCR was performed in a final volume of 20 μL containing 10 μL of TaqMan Universal Master Mix (Applied Biosystems, Foster City, CA, USA), 900 nM of each of the forward and reverse primers (Bio-Rad Laboratories, Hercules, CA, USA), 250 nM of the probe (Bio-Rad Laboratories), and 100 ng of the DNA sample. The concentration of the DNA samples was determined by Nano Vue Plus (GE HealthCare, Boston, MA, USA). The following primers and probes were used for the detection of four BPV genotypes.

Four separated PCR reactions were performed on the CFX96 Real-Time System of the C1000 Touch Thermal Cycler (Bio-Rad Laboratories)^7,19^. The thermal cycling conditions were as follows: 50 °C for 2 min, 95 °C for 10 min, and 40 cycles of 95 °C for 15 s and 58 °C for 60 s. Each sample was analyzed in duplicate, and negative controls were included in all runs. Data acquisition and data analyses were performed using CFX Maestro (Bio-Rad Laboratories) software. The same samples used as positive controls for ddPCR were also tested using qPCR.

### DdPCR

For ddPCR, Bio-Rad’s QX100 ddPCR System was used according to the manufacturer’s instructions. The reaction was performed in a final volume of 20 μL containing 10 μL of ddPCR Supermix for Probes (no dUTP 2 × ; Bio-Rad), 0.9 μM primer, and 0.25 μM probe (Table 1) with 5 μL sample DNA (100 ng). A black hole quencher was used in combination with FAM and VIC fluorescent dye reporters (Bio-Rad Laboratories). The reaction mixture was placed into the sample well of a DG8 cartridge (Bio-Rad Laboratories). A volume of 70 μL of droplet generation oil was loaded into the oil well, and droplets were formed in the droplet generator (Bio-Rad Laboratories). After processing, the droplets were transferred to a 96-well PCR plate (Eppendorf, Hamburg, Germany). PCR amplification was carried out on a T100 Thermal Cycler (Bio-Rad Laboratories) with the following thermal profile: hold at 95 °C for 10 min, 40 cycles of 94 °C for 30 s and 58 °C for 1 min, 1 cycle at 98 °C for 10 min, and ending at 4 °C. After amplification, the plate was loaded onto a droplet reader (Bio-Rad Laboratories) and the droplets from each well of the plate were read automatically. QuantaSoft software was used to count the PCR-positive and PCR-negative droplets to provide absolute quantification of the target DNA. Therefore, the ddPCR results could be directly converted into copies/µL in the initial samples simply by multiplying the concentration (copies/µL) obtained from QuantaSoft software by 20 µL, that is the total volume of the reaction mixture. That number was then divided by 5 µL, that is the volume of DNA sample added to the reaction mixture at the beginning of the assay. Each sample was analyzed in duplicate. Samples with very few positive droplets were re-analyzed to ensure that these low copy number samples were not due to cross-contamination.

**Table 1:** Primers and probes used for detection of BPV-1, -2, -13, and -14 in ddPCR and qPCR.

	Forward 5′ 3'	Reverse 5′ 3'	Probe	Region	Size- bp
BPV1	ACTTCTGATCACTGCCATT	ATAGAAACCATAGATTTGGCA	TGAAGTGTTTCTGTTTGTGA FAM	E5 3'UTR/ORF E5	67
BPV2	TACAGGTCTGCCCTTTTAAT	ACAGTAAACAAATCAAATCCA	AACAACAAAGCCAGTAACC VIC	ORF E5/E5 5'UTR	77
BPV13	CTGTGTGGATTTGATTTGTT	CAGGGGGAATACAAATTCT	TGAAGTGTTTCTGTTTGTGA FAM	E5 5' UTR	98
BPV14	CTTTGTTATTGTATATGAGTCTGT	ACTCTTGACGGTTTAAAAGTA	ATCTTGCCAGTGATCCTG FAM	E5 5' UTR	98

### PCR

PCR analysis was carried out on all sheep blood samples to detect DNA of OaPV3 and OaPV4, the only OaPVs reported in Italy to date^20,21^. PCR was carried out in a final volume of 25 μL containing 12.5 μL of EconoTaq PLUS 2 × Master Mix (Lucigen, Middleton, WI, USA), 1 mM of each of the forward and reverse primers and 100 ng of the DNA sample, extracted as before reported. The following primers were used: OaPV3, L1 forward: AACGGACTTGTCTTCCATG; OaPV3, L1 reverse: AAAGACTCGGTATTGGGAGGT; OaPV3, E6 forward: AAGCCCTCGTACAATAGCTG; OaPV3, E6 reverse: GCCAAATCTCCAGAGTAAAGC; OaPV4, E6 forward: CCAAGATGCTGAGCAGTAAATTCC; OaPV4, E6 reverse: TTATGGCTATTTGGTCCGTG. The positive control tissues for OaPV3 and OaPV4 were samples from ovine ocular squamous cell carcinoma and ovine cutaneous fibropapilloma, respectively, (a kind gift from prof. A. Alberti, Department of Veterinary Medicine of Sassari University). The reaction was performed on the T100 Thermal Cycler (Bio-Rad Laboratories) and the cycling conditions were as follows: 95 °C for 2 min, and 40 cycles of 95 °C for 30 s, 56 °C for 30 s and 72 °C for 30 s. For each sample, the PCR experiment was repeated twice to validate the accuracy of the obtained data.

### Statistical analysis

Differences in the proportions of detected cases were tested using the chi-square test by Campbell and Richardson^22^. Furthermore, regarding the significance relative to the number of copies of BPV DNA detected in sheep in the different regions, the t-test was used after adjusting for the Bonferroni multiple comparison correction of means. *P*-values ≤ 0.05 were considered to be statistically significant. All analyses were performed using R statistical software (version 4.0.3; The R Foundation, Vienna, Austria).

### Ethics statement

Blood samples were collected from animals in public slaughterhouses during the mandatory ante-mortem clinical examination. All procedures performed in this study followed common good clinical practices and received institutional approval from the Ethical Animal Care and Use Committee of the University of Naples Federico II (PG/2017/0099607). All farmers were previously informed and in agreement with the purpose and methods used.

## Results

**Figure 1:**
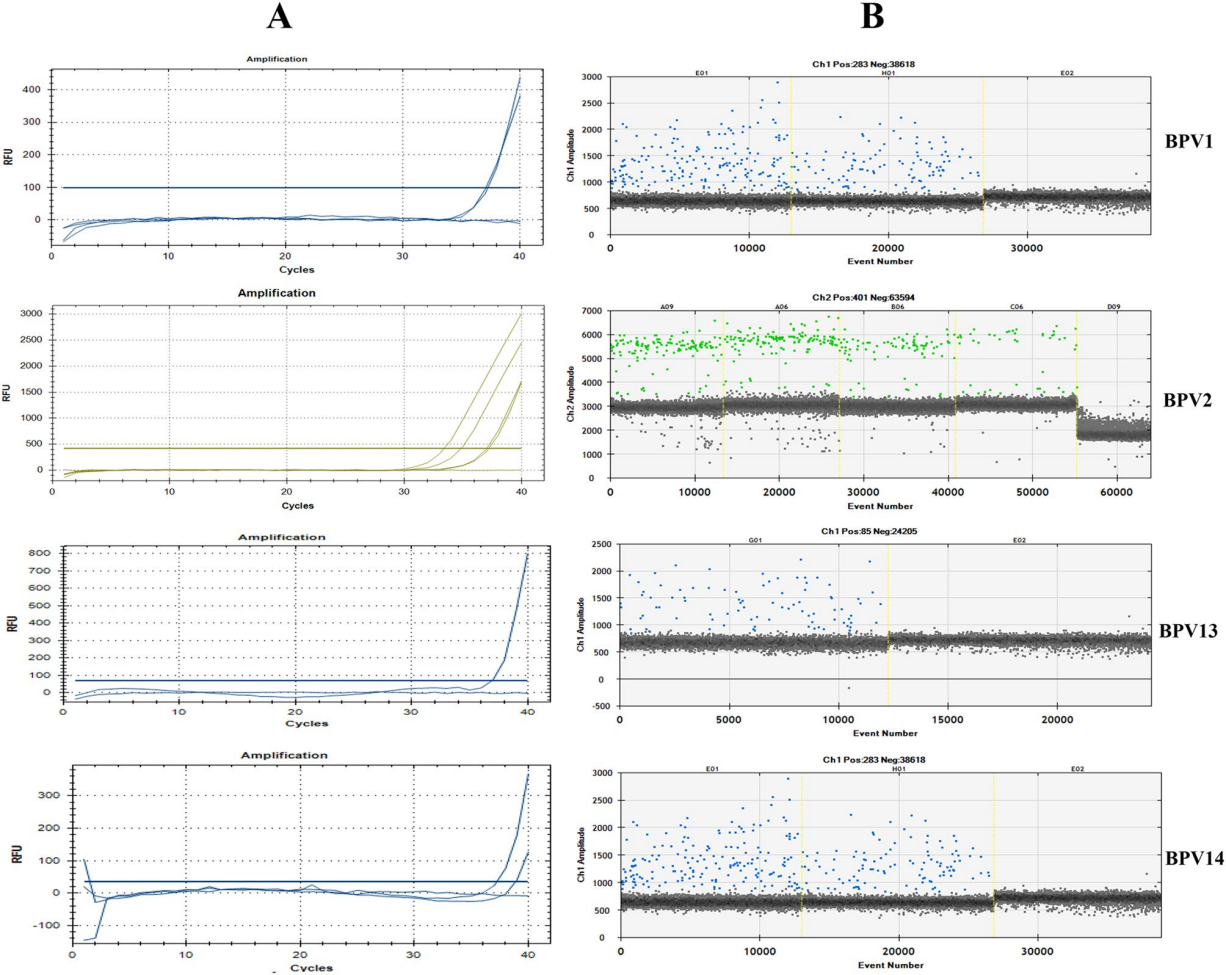
qPCR curves (**A**) and the relative rain plots of the ddPCR (**B**) for the four BPVs. For BPV1 two positive and one negative samples; for BPV2 four positive and one negative samples; for BPV13 one positive and one negative sample and for BPV14 two positive and one negative samples are shown, respectively.

Overall, our results showed that BPV DNA was found in 68 out of 103 blood samples (66%) from healthy sheep using ddPCR. The same liquid biopsies were also investigated using qPCR, which revealed BPV DNA in approximately 9% of blood samples. Figure 1 reported the cycle of quantification (Cq) for the qPCR results for both the positive and negative samples. Data from qPCR were compared to ones obtained by ddPCR performed on the same samples correlated Cq and copy number obtained by qPCR and ddPCR, respectively (Supplemental Fig. S1). In particular, ddPCR detected and quantified BPV-1 DNA in 9 samples compared to qPCR that was able to detect BPV-1 DNA in 2 samples; in 51 blood samples, BPV-2 DNA was revealed by ddPCR in comparison with only 4 samples revealed by qPCR. Furthermore, BPV-13 DNA was revealed in 22 blood samples by ddPCR whereas qPCR was able to detect only one sample. Finally, ddPCR revealed the presence of BPV-14 DNA in 14 blood samples compared to 4 samples found positive by qPCR.

**Figure 2:**
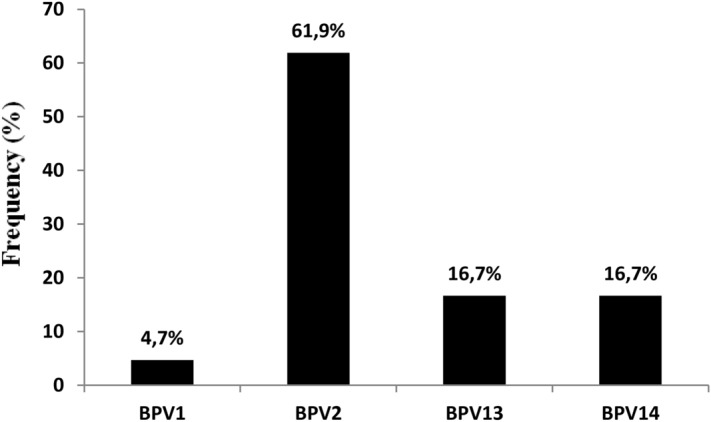
Percentages of single BPV DNA detection found in the 42 positive samples.

In 42 of the positive samples (61.8%), a single BPV infection was observed, 26 of which were caused by BPV-2 (61.9%) and 7 by BPV-13 (16.7%). BPV-14 was responsible for 7 single infections (16.7%), and BPV-1 for two single infections (4.7%) (Fig. 2). Multiple BPV infections were seen in 26 (38.2%) positive samples. BPV coinfections caused by two genotypes were seen in 22 positive cases (84.6%), with dual BPV-2/BPV-13 infection being the most prevalent as it was seen in 10 blood samples. Furthermore, dual coinfections were also detected such as seven BPV-2/BPV-14, four BPV-1/BPV2, and only one BPV-1/BPV-13. BPV coinfections by triple and quadruple genotypes were detected in 11.5% (3/26) and 3.8% (1/26) of blood positive samples as reported in the Table 2.

**Table 2:** Genotype coinfections with related number of their combination are shown.

Coinfections	Genotype combinations	Number
Double	BPV2/BPV13	10
	BPV2/BPV14	7
	BPV1/BPV2	4
	BPV1/BPV13	1
Triple	BPV1/BPV2/BPV13	1
	BPV2/BPV13/BPV14	2
Quadruple	BPV1/BPV2/BPV13/BPV14	1

Double and triple coinfections based on other genotype combinations were not detected.

In sheep flocks that lived and shared lands with cattle, BPV DNA was detected in approximately 53% of blood samples collected in Apu (8/15), 75% of samples acquired in both Bas and Cal (18/24), and 100% of blood samples harvested from Sar (20/20). In sheep flocks from Cam that lived in isolated and closed pens without any contact with cattle, BPV DNA was detected in 20% of blood samples examined (4/20). The percentage differences in BPV infections in all sheep flocks with cattle contact were statistically significant compared to the percentage observed in sheep flocks without any contact with cattle, as the Campbell-Ricardson’s chi-square test resulted in a *p*-value < 0.05. Furthermore, in all geographical areas except for Apu, BPV-2 was the most prevalent genotype. BPV-13 and BPV-14, as well as BPV-1 were also observed. Furthermore, Apu BPV-14 showed the highest number of copies/µL; it was the most prevalent BPV genotype at a detection level of 40% in the examined samples (6/15) and a statistically significant Campbell-Ricardson’s chi-square test *p*-value < 0.05.

The overall quantification results showed that viral copy numbers/µL ranged from 0.38 to 18.9 for BPV-1, 0.32 to 21.48 for BPV-2, 0.32 to 18.56 for BPV-13, and 0.38 to 18.32 for BPV-14 (Supplemental Table S1). Using t-tests, the differences between the copy numbers of BPV-2 in Sar compared to the other means found in Cal, Cam, Bas, and Apu were statistically significant with *p*-values < 0.05. Indeed, after adjusting for the Bonferroni multiple comparison correction, their *p*-values were 0.003 (Sar-Cal), 0.04 (Sar-Cam), 0.002 (Sar-Bas), and 0.01 (Sar-Apu), respectively.

Neither OaPV3 nor OaPV4 DNA was detected in all blood samples.

## Discussion

To our knowledge, this study is the first to use the sensitive ddPCR assay, a biotechnological refinement of conventional PCR, to detect and quantify highly pathogenic bovine δPVs in clinically healthy sheep by liquid biopsy. Our previous studies have shown that ddPCR has both high specificity and sensitivity for the detection and quantification of BPV DNA in healthy and diseased cattle^7^ as well as in healthy goats^19^. The current study provides further evidence that ddPCR is a very useful approach to detect and quantify BPV in the blood of healthy sheep and allows us to gain diagnostic and epidemiological insights into BPV presence in ovine species as data on the prevalence and types of BPVs in sheep are not currently available.

The liquid biopsy approach to the detection of circulating BPV DNA has garnered growing interest in PV studies^23^. Indeed, PV detection in the blood can be used as diagnostic, prognostic, and epidemiological markers^24^.

Our study showed that BPV-2 is the most prevalent BPV genotype in healthy sheep, similar to other ruminants, such as cattle and goats. The highest number of copies of this genotype was found in Sar from sheep flocks in which cross-infections by BPVs have been previously reported^13^, which suggests that copy numbers may correlate with the risk of cutaneous and mucosal lesions that progress to cancer. Furthermore, this study reports the first detection of BPV-14 in sheep. This genotype, chronologically the last BPV type identified in cattle, has never been described in the ovine species. Furthermore, our results demonstrated a statistically significant prevalence of BPV-14 in the flocks from Apu compared to flocks from Sar, Cal, Bas, and Cam, which suggested that it is conceivable that BPV genotype prevalence has a territorial divergence in these regions.

We compared the sensitivity of ddPCR with that of qPCR in evaluating the same liquid biopsy, demonstrating that ddPCR has superior sensitivity compared to qPCR. Therefore, our results suggest that ddPCR is by far the most sensitive and accurate assay for BPV detection. It is worth noting that it has been shown that ddPCR outperforms qPCR in terms of the sensitivity, specificity, and reproducibility of oncogenic human papillomavirus detection and quantification^25–27^.

BPV genotype detection in the blood of healthy sheep suggests that the bloodstream can be the primary site of BPV infection. As bovine δPVs are known to infect ovine species and result in anatomoclinical diseases, it is conceivable that these viruses may spread through the blood, which could be responsible for secondary tissue localization and infection. However, as in humans^1^, further epidemiological studies are required to enhance the understanding of BPV transmission via the bloodstream.

Here, we detected a higher percentage of BPVs in sheep that were in close contact with cattle herds, about which numerous case reports of BPV infection have been described. Indeed, the sheep flocks in our study shared bracken fern-infested lands with pasture-residing cattle for grazing^12^. The evidence from epidemiological studies of cattle is sufficiently strong to suggest that in the presence of BPV infection, the toxic components of bracken ferns such as ptaquiloside (PT), a water-soluble norsesquiterpenoid glycoside, are ecological co-factors in the development of severe diseases due to BPVs, including chronic enzootic hematuria (CEH), a clinical syndrome caused by bladder tumors^28^. Thus, it is conceivable that PT may also be a co-factor of diseases in sheep. Indeed, PT has recently been detected in biological matrices from healthy sheep^29^. PT is known to hamper the immune system and may play an important role in cross-species transmission and infection of bovine δPVs. It is worth noting that outbreaks of CEH have also been reported in sheep^30,31^. Furthermore, bovine δPV infection resulting in clinical disease are known to occur in sheep^10–12^. Therefore, the detection of bovine δPV DNA in the blood of sheep means that sheep can be infected by these PVs, which may make δPVs an additional, potential cause of ovine disease. Furthermore, our results suggest that clinically healthy sheep may represent a reservoir for bovine δPVs. Thus, it is conceivable that sheep may play a role in intra- and interspecies bovine δPV transmission and infection. In this context, very precise quantitation of very low viral copy numbers can provide more precise monitoring of latent BPV DNA reservoirs.

Finally, it is well known that PV distribution varies considerably by geography^32^. Therefore, ddPCR may be an essential tool for improving diagnostic procedures thus allowing the identification of the genotypic distribution of BPV and a better understanding of the possible, geographical divergence of BPV prevalence in different areas. The ddPCR assay appears to possess high sensitivity and accuracy, which is valuable for addressing the molecular burden of BPV infections and useful for defining an accurate ecological epidemiology. This baseline information improves our knowledge about the molecular mechanisms of the disease and provides insights into necessary measures for reducing the risk of BPV infection and/or co-infection.

In conclusion, ddPCR is presently being used to detect very low nucleic acid concentrations and, therefore, appears to be of interest in the diagnosis of infectious diseases, including viral diseases^33^. DdPCR has proven to be a valuable new technology and with additional improvements in prospect it is likely to become an indispensable tool in diagnostic, prognostic and epidemiological virus research^18^. Therefore, the ddPCR method may provide a new and promising tool for evaluating the BPV viral load in clinical samples. Future PV research warrants the use of this molecular approach to assess PV type-specific pathogenetic pathways of disease, including carcinogenicity. Indeed, available evidence from BPV distribution lends strong support to the notion that the risk of an animal developing a BPV-associated disease varies substantially according to the specific BPV type with which the animal is infected. Finally, the ddPCR approach may provide a better understanding of the complex interactions between multiple BPV types during coinfections, as the possible interference resulting from multiple PV genotypes in coinfection cases remains an open question^34^.

## Supplementary Information

Supplementary Figure S1.

Supplementary Table S1.

## Data Availability

All data generated and/or analyzed during the current study are available from the corresponding author on reasonable request.

## Abbreviations

Apu: Apulia
Bas: Basilicata
BPV: Bovine papillomavirus
Cal: Calabria
Cam: Campania
CEH: Chronic enzootic hematuria
ddPCR: Digital droplet polymerase chain reaction
δPV: *Delta* Papillomavirus
dUTP: 2’-Deoxyuridine, 5’-Triphosphate
EDTA: Ethylenediaminetetraacetic acid
FAM: Fluorescein amidite
PCR: Polymerase chain reaction
PT: Ptaquiloside
PV: Papillomavirus
qPCR: Real time quantitative polymerase chain reaction
Sar: Sardinia
VIC: Victoria
