# Supplementary figures and images for: Detection and quantification of bovine papillomavirus DNA by digital droplet PCR in sheep blood
Source: Sci Rep. 2021 May 13;11:10292. doi: 10.1038/s41598-021-89782-4 (PMC8119674; doi:10.1038/s41598-021-89782-4)

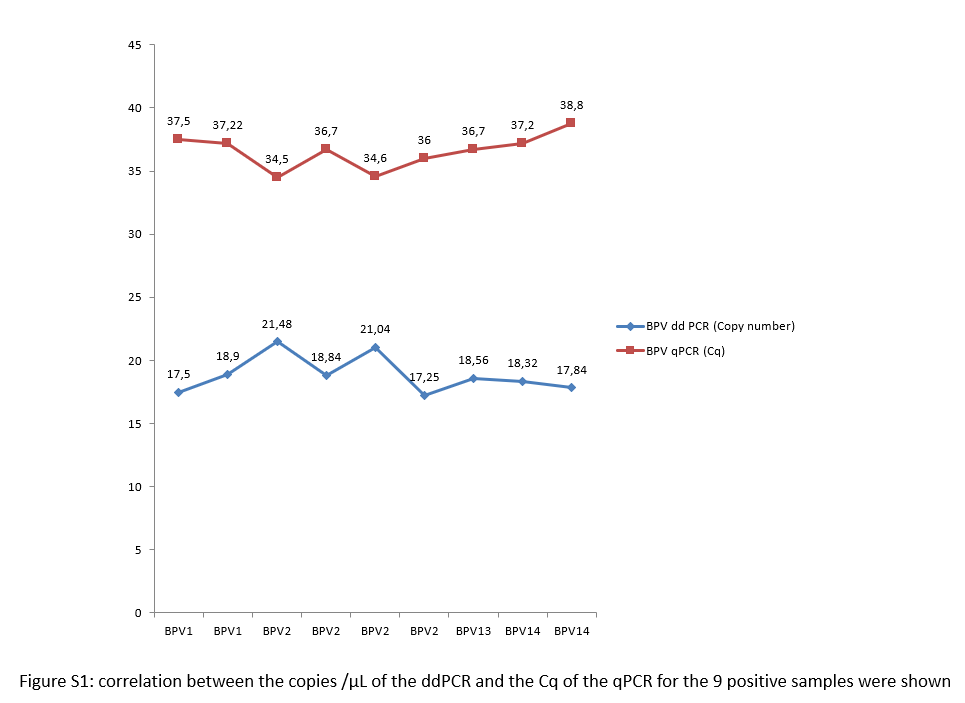

Supplement: Supplementary file 1 — Supplementary Figure S1. [file 41598_2021_89782_MOESM1_ESM.tif]
