# Supplementary material for: Detection and quantification of bovine papillomavirus DNA by digital droplet PCR in sheep blood
Source: Sci Rep. 2021 May 13;11:10292. doi: 10.1038/s41598-021-89782-4 (PMC8119674; doi:10.1038/s41598-021-89782-4)
Supplement: Supplementary file 2 — Supplementary Table S1. [file 41598_2021_89782_MOESM2_ESM.docx]

**Table S1 shows the copy number obtained in ddPCR and the Cq in qPCR for each of the 103 samples. All analyses were performed using R statistical software (4.0.3: The R Foundation, Vienna, Austria)**

| **Region** | **sample** | **BPV 1 ddPCR copy number** | **BPV1 qPCR Cq** | **BPV 2 ddPCR copy number** | **BPV2qPCR Cq** | **BPV 13 ddPCR copy number** | **BPV13qPCR Cq** | **BPV 14 ddPCR copy number** | **BPV14qPCR Cq** |
| --- | --- | --- | --- | --- | --- | --- | --- | --- | --- |
| **Sardinia** | P1 | N | N | 21,48 | 34,5 | N | N | N | N |
|  | P2 | N | N | 1,092 | N | 0,34 | N | N | N |
|  | P3 | N | N | 18,84 | 36,7 | N | N | N | N |
|  | P4 | N | N | 3,9 | N | N | N | N | N |
|  | P5 | N | N | 9,04 | N | 0,74 | N | 1,7 | N |
|  | P6 | N | N | 12,5 | N | N | N | N | N |
|  | P7 | N | N | 21,04 | 34,6 | 3,7 | N | N | N |
|  | P8 | 17,5 | 37,05 | 17,25 | 36 | N | N | N | N |
|  | P9 | N | N | 2,804 | N | N | N | N | N |
|  | P10 | N | N | 1,812 | N | N | N | N | N |
|  | P11 | N | N | 4,92 | N | N | N | N | N |
|  | P12 | N | N | 1,324 | N | N | N | N | N |
|  | P13 | N | N | 2,732 | N | 0,41 | N | N | N |
|  | P14 | N | N | 2,916 | N | N | N | N | N |
|  | P15 | N | N | 2,892 | N | N | N | N | N |
|  | P16 | N | N | 4,712 | N | N | N | 3 | N |
|  | P17 | N | N | 1,258 | N | 1,2 | N | N | N |
|  | P18 | N | N | 0,5286 | N | N | N | N | N |
|  | P19 | N | N | 2,42 | N | N | N | 1,1 | N |
|  | P20 | N | N | 1,367 | N | N | N | N | N |
| **Calabria** | P21 | 2,49 | N | 0,3912 | N | N | N | N | N |
|  | P22 | N | N | 0,3512 | N | N | N | N | N |
|  | P23 | N | N | N | N | N | N | N | N |
|  | P24 | N | N | 0,36 | N | N | N | N | N |
|  | P25 | 18,9 | 37,22 | N | N | 18,56 | 36,7 | N | N |
|  | P26 | N | N | N | N | N | N | N | N |
|  | P27 | N | N | N | N | 3,67 | N | N | N |
|  | P28 | N | N | N | N | N | N | N | N |
|  | P29 | N | N | 1,39 | N | 0,33 | N | N | N |
|  | P30 | N | N | N | N | 1,37 | N | N | N |
|  | P31 | N | N | 0,74 | N | 0,86 | N | N | N |
|  | P32 | N | N | 1,16 | N | 0,39 | N | N | N |
|  | P33 | N | N | N | N | N | N | N | N |
|  | P34 | 0,78 | N | 1,05 | N | 1,17 | N | N | N |
|  | P35 | 0,44 | N | 0,71 | N | N | N | N | N |
|  | P36 | N | N | N | N | N | N | N | N |
|  | P37 | N | N | N | N | 2,35 | N | N | N |
|  | P38 | 0,38 | N | N | N | N | N | N | N |
|  | P39 | N | N | 0,82 | N | N | N | N | N |
|  | P40 | N | N | N | N | N | N | N | N |
|  | P41 | 1,17 | N | 0,56 | N | 0,32 | N | 2,8 | N |
|  | P42 | N | N | 1,52 | N | N | N | N | N |
|  | P43 | N | N | 0,63 | N | N | N | N | N |
|  | P44 | N | N | 0,77 | N | N | N | N | N |
| **Campania** | P45 | N | N | N | N | N | N | N | N |
|  | P46 | N | N | N | N | N | N | N | N |
|  | P47 | N | N | N | N | N | N | N | N |
|  | P48 | N | N | N | N | N | N | N | N |
|  | P49 | N | N | N | N | N | N | N | N |
|  | P50 | N | N | N | N | N | N | N | N |
|  | P51 | N | N | N | N | N | N | 0,8 | N |
|  | P52 | N | N | N | N | N | N | N | N |
|  | P53 | N | N | N | N | N | N | N | N |
|  | P54 | N | N | N | N | N | N | N | N |
|  | P55 | N | N | N | N | N | N | N | N |
|  | P56 | N | N | N | N | N | N | N | N |
|  | P57 | N | N | N | N | N | N | N | N |
|  | P58 | N | N | N | N | N | N | N | N |
|  | P59 | N | N | N | N | N | N | N | N |
|  | P60 | N | N | N | N | 1,44 | N | N | N |
|  | P61 | N | N | N | N | N | N | N | N |
|  | P62 | N | N | N | N | N | N | N | N |
|  | P63 | N | N | 1,16 | N | N | N | N | N |
|  | P64 | N | N | 3,29 | N | N | N | 2,18 | N |
| **Basilicata** | P65 | N | N | N | N | 0,41 | N | N | N |
|  | P66 | N | N | 0,36 | N | N | N | N | N |
|  | P67 | N | N | 1,02 | N | N | N | 0,84 | N |
|  | P68 | N | N | 0,81 | N | 0,84 | N | N | N |
|  | P69 | N | N | 0,57 | N | N | N | N | N |
|  | P70 | N | N | N | N | 0,66 | N | N | N |
|  | P71 | N | N | 0,72 | N | N | N | N | N |
|  | P72 | N | N | N | N | N | N | N | N |
|  | P73 | N | N | 0,35 | N | N | N | 0,38 | N |
|  | P74 | N | N | N | N | N | N | 0,38 | N |
|  | P75 | N | N | 0,47 | N | 0,48 | N | 0,46 | N |
|  | P76 | N | N | N | N | N | N | 0,38 | N |
|  | P77 | N | N | 0,36 | N | 0,84 | N | N | N |
|  | P78 | N | N | N | N | N | N | N | N |
|  | P79 | 0,92 | N | 0,51 | N | N | N | N | N |
|  | P80 | N | N | 0,37 | N | N | N | N | N |
|  | P81 | N | N | 1,07 | N | N | N | N | N |
|  | P82 | N | N | N | N | N | N | N | N |
|  | P83 | N | N | N | N | N | N | N | N |
|  | P84 | 0,47 | N | N | N | N | N | N | N |
|  | P85 | N | N | N | N | N | N | N | N |
|  | P86 | N | N | 0,68 | N | 0,85 | N | N | N |
|  | P87 | N | N | 0,54 | N | N | N | N | N |
|  | P88 | N | N | N | N | N | N | N | N |
| **Apulia** | p89 | N | N | N | N | N | N | 18,32 | 37,2 |
|  | p90 | N | N | N | N | N | N | 17,84 | 38,8 |
|  | p91 | N | N | N | N | N | N | 2,82 | N |
|  | p92 | N | N | 0,32 | N | N | N | 4,6 | N |
|  | p93 | N | N | N | N | N | N | N | N |
|  | p94 | N | N | 0,72 | N | N | N | 1,48 | N |
|  | p95 | N | N | N | N | N | N | 0,8 | N |
|  | p96 | N | N | N | N | 0,43 | N | N | N |
|  | p97 | N | N | N | N | N | N | N | N |
|  | p98 | N | N | N | N | N | N | N | N |
|  | p99 | N | N | N | N | N | N | N | N |
|  | p100 | N | N | N | N | N | N | N | N |
|  | p101 | N | N | 0,37 | N | N | N | N | N |
|  | p102 | N | N | N | N | N | N | N | N |
|  | p103 | N | N | N | N | N | N | N | N |
